# Assembly of Supramolecular Nanoplatelets with Tailorable Geometrical Shapes and Dimensions

**DOI:** 10.3390/polym15112547

**Published:** 2023-05-31

**Authors:** Moyan Wang, Gangfeng Chen, Xiaojian Hou, Yunjun Luo, Bixin Jin, Xiaoyu Li

**Affiliations:** 1 Experimental Center of Advanced Materials, School of Materials Science and Engineering, Beijing Institute of Technology, No.5 Zhongguancun South St., Beijing 100081, Chinahou18810329135@163.com (X.H.);; 2 Key Laboratory of High Energy Density Materials, MOE, Beijing Institute of Technology, No.5 Zhongguancun South St., Beijing 100081, China

**Keywords:** host–guest, nanoplatelet, cyclodextrin

## Abstract

The craving for controllable assembly of geometrical nanostructures from artificial building motifs, which is routinely achieved in naturally occurring systems, has been a perpetual and outstanding challenge in the field of chemistry and materials science. In particular, the assembly of nanostructures with different geometries and controllable dimensions is crucial for their functionalities and is usually achieved with distinct assembling subunits via convoluted assembly strategies. Herein, we report that with the same building subunits of α-cyclodextrin (α-CD)/block copolymer inclusion complex (IC), geometrical nanoplatelets with hexagonal, square, and circular shapes could be produced by simply controlling the solvent conditions via one-step assembly procedure, driven by the crystallization of IC. Interestingly, these nanoplatelets with different shapes shared the same crystalline lattice and could therefore be interconverted to each other by merely tuning the solvent compositions. Moreover, the dimensions of these platelets could be decently controlled by tuning the overall concentrations.

## 1. Introduction

Two-dimensional (2D) nanomaterials have instigated enormous curiosity because of their unique physicochemical properties such as unprecedented electronic properties due to their ultrathin nanoscale thickness and flat morphology [1,2,3]. In recent years, 2D platelets have been applied in numerous fields and their unique aspect ratios have been demonstrated to be crucial for their applications, such as catalysis [4,5], biological nanomaterials [6,7], sensing [8,9], and optoelectronic devices [10,11]. However, the fabrication of uniform and regular 2D platelets with tunable shapes and sizes remains challenging. To achieve such excellent 2D architectures in nanoscales, living seeded self-assembly has stood out from many approaches as a powerful strategy for the fabrication of diverse 2D architectures with controlled shapes and sizes [12]. In particular, the most powerful and facile method, crystallization-driven self-assembly (CDSA) of block copolymers (BCP), which was pioneered by Manners, Winnik, and coworkers [13,14,15,16], has been applied to fabricate uniform fibrillar micelles and 2D platelets with different shapes. Despite the excellent efficiency and controllability of this approach, it is limited to a few specific crystalline polymers, of which the geometry of the resultant platelets is mainly decided and confined by the lattice structure of their unit cell. Moreover, the preparation of self-assembled platelets normally requires multi-step preparation, unideal for scalable production and application.

In the meantime, studies on inclusion complexes (ICs) from supramolecular chemistry systems have flourished in recent years, as they can be exploited as models for molecular recognition for applications [17,18,19,20]. In particular, ICs could be fabricated by threading host molecules with guest polymer chains [21,22]. As the most representative host molecules, cyclodextrin (CD) is widely studied because of its easy preparation, low-cost, and biocompatibility. CDs are cyclic oligomers constructed from six, seven, or eight 1,4-linked glucose units (referred to as α-, β-, and γ-CD, respectively), and can form ICs with guest polymers [23]. The three-dimensional structure of CD molecules is hollow truncated cones, giving them a cavity for molecular recognition. The cavity depth of CD is about 7.8 Å, and for α-, β-, and γ-CD, the inner diameters of the primary hydroxyl end are about 4.5, 7.0, and 8.5 Å, respectively, which are smaller than the secondary hydroxyl end. In addition, the outer edge of CD molecules is hydrophilic, while the inner cavity is hydrophobic, allowing for the inclusion of appropriately sized guest molecules through hydrophobic interactions. The matching of the polymer chain cross-sectional area with the CD cavity diameter is a key factor to form ICs. For instance, poly(ethylene oxide) (PEO) can form ICs with α-CD [24] but not β- or γ-CD [25], while poly(propylene oxide) (PPO) can form ICs with β- and γ-CD but not α-CD [26,27]. The ICs can further assemble into well-defined structures based on hydrogen bonding interactions between the outer cavities [28,29]. Since the first report of ICs from α-CD and PEO in the 1990s [30], numerous studies have been reported on the fabrication of nanostructures from ICs [31], yet the tunability of sizes and shapes has been difficult to realize.

**Figure 1:**
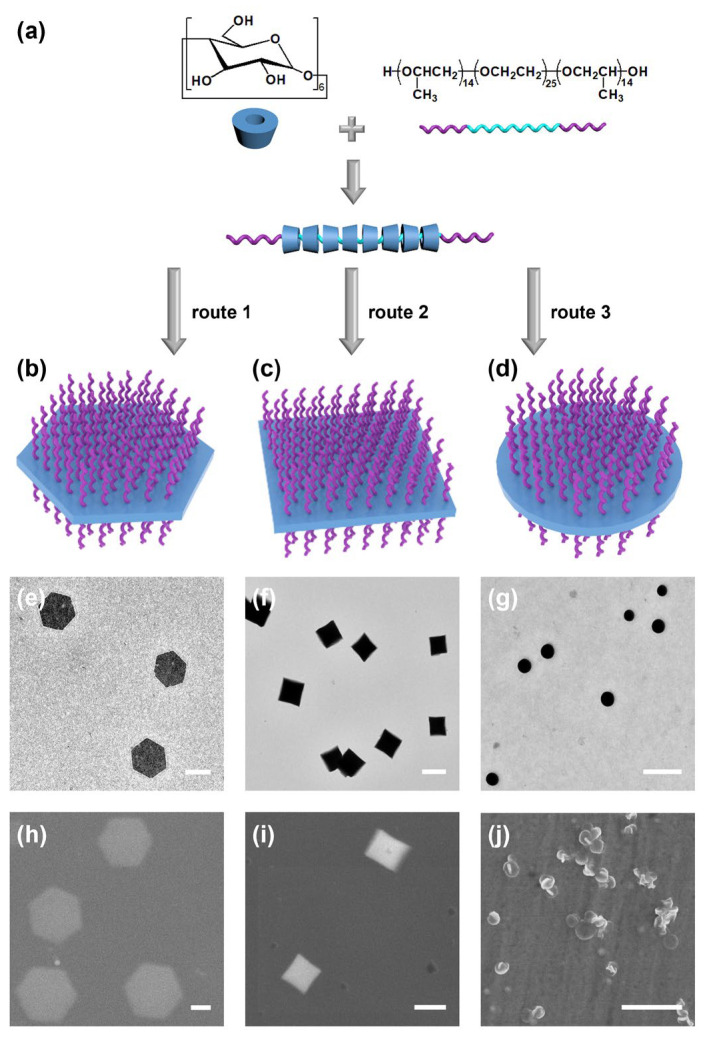
Chemical structures and schematic illustration of the (**a**) co-assembly process of P-E-P triblock copolymer and α-CD. Schematic illustrations, and the corresponding TEM and SEM images of the assembled platelets from P-E-P and α-CD in the solvent mixtures of (**b**,**e**,**h**) 2-PrOH:H_2_O = 1:1; (**c**,**f**,**i**) H_2_O:toluene = 1:1; and (**d**,**g**,**j**) 2-PrOH:H_2_O:toluene = 1:1:0.3. Scale bars are 1 μm.

In our previous work, two-dimensional hexagonal platelets were fabricated through the formation of crystalline ICs of the *tris*-*o*-phenylenedioxycyclotriphosphazene (TPP) host and the block copolymer guest, and with well-defined geometrical shape [32]. Moreover, hexagonal platelets with uniform size were formed through a seeded growth process, and their dimensions were tunable by adjusting the mass ratio between the seeds and TPP/copolymer mixture. This study reported the preparation of two-dimensional materials with controllable dimensions based on the host–guest interaction for the first time, but the adjustability of their geometrical shape has not been achieved yet. In this work, we present a supramolecular strategy to prepare 2D platelets with variable shapes and uniform sizes from the ICs of α-CD and PPO_14_-*b*-PEO_25_-*b*-PPO_14_ (P-E-P) triblock copolymers in a one-step procedure (Figure 1). α-CD could thread into the PEO block and form ICs, which subsequently crystallized to produce nanoplatelets with PPO as the stabilizing corona. By carefully controlling the solvent compositions, 2D platelets with variable shapes were fabricated, including hexagonal, square, and circular. The sizes of the nanoplatelets were quite uniform and positively correlated with the solution concentrations. Moreover, the shapes of these nanoplatelets were interconvertible by simply changing solvent compositions.

## 2. Materials and Methods

### 2.1. Materials

Poly(propylene oxide)-*b*-poly(ethylene oxide)-*b*-poly(propylene oxide) (PPO-*b*-PEO-*b*-PPO, *M_n_*~2700 g/mol) triblock copolymer, α-cyclodextrin (α-CD, 98%), and other chemicals were purchased from Aldrich (St. Louis, MO, USA), and were used as received.

### 2.2. Methods

#### 2.2.1. Preparation of Hexagonal Platelets, Square Platelets and Circular Platelets of P-E-P/𝛼-CD Inclusion Complex

To obtain the hexagonal platelets of P-E-P/𝛼-CD inclusion complex, 𝛼-CD solid and P-E-P were mixed at an EO/𝛼-CD molar ratio of 1:1 and dispersed in 2 mL mixture of H_2_O/2-PrOH (1:1, volume ratio). The solution was heated at 80 °C with a stirring rate of 800 r/min for 1 h before the solution was allowed to cool down naturally to 20 °C.

To obtain the square platelets of P-E-P/𝛼-CD inclusion complex, 𝛼-CD solid and P-E-P were mixed at an EO/𝛼-CD molar ratio of 1:1 and dispersed in 2 mL mixture of H_2_O/toluene (1:1, volume ratio). The solution was heated at 80 °C with a stirring rate of 800 r/min for 1 h before the solution was allowed to cool down naturally to 20 °C.

To obtain the circular platelets of P-E-P/𝛼-CD inclusion complex, 𝛼-CD solid and P-E-P were mixed at an EO/𝛼-CD molar ratio of 1:1 and dispersed in 2 mL mixture of H_2_O/2-PrOH/toluene (1:1:0.3, volume ratio). The solution was heated at 80 °C with a stirring rate of 800 r/min for 1 h before the solution was allowed to cool down naturally to 20 °C.

Dry the solution of geometrical platelets after centrifugation and weigh the gained powder. The yields of hexagonal, square, and circular plates obtained at fixed assembly concentrations were calculated to be 74.7%, 61.3%, and 64.7%, respectively.

#### 2.2.2. Interconversion of Hexagonal Platelets, Square Platelets and Circular Platelets

To achieve the morphology conversion from hexagonal platelets to circular platelets, the hexagonal platelets solution obtained from the last section and the fresh toluene were mixed evenly at a volume ratio of 1:0.15 at room temperature.

To achieve the morphology conversion from square platelets to circular platelets, the aqueous phase of the square platelet solution and the desired amount of fresh 2-PrOH and toluene were mixed at a volume ratio of 1:1:0.3 at room temperature.

To achieve the morphology conversion from square platelets to hexagonal platelets, the aqueous phase of the square platelet solution and the desired amount of fresh 2-PrOH were mixed at a volume ratio of 1:1 at room temperature.

To achieve the morphology conversion from circular platelets to hexagonal platelets, the circular platelet solution was dried in a vacuum oven at 40 °C for 24 h; then, the dry powders were dispersed in 2 mL mixture of H_2_O/2-PrOH (1:1, volume ratio) at room temperature.

To achieve the morphology conversion from circular platelets to square platelets, the circular platelet solution was dried in a vacuum oven at 40 °C for 24 h; then, the dry powders were dispersed in 2 mL mixture of H_2_O/toluene (1:1, volume ratio) at room temperature.

To achieve the morphology conversion from hexagonal platelets to square platelets, the hexagonal platelet solution obtained from the last section was dried in a vacuum oven at 40 °C for 24 h; then, the dry powders were dispersed in 2 mL mixture of H_2_O/toluene (1:1, volume ratio) at room temperature.

#### 2.2.3. Transmission Electron Microscopy (TEM)

The samples for electron microscopy were prepared by drop-casting one drop (~7 µL) of the solution onto a carbon coated copper grid. Grids were placed on a piece of filter paper in advance to quickly remove excess solvent in 1 s to prevent further morphological change.

#### 2.2.4. Scanning Electron Microscopy (SEM)

SEM experiments were conducted directly on the carbon-coated copper grid used for TEM analysis. An ultrathin coating of Au (~5 nm) was deposited via high vacuum evaporation.

#### 2.2.5. Wide-Angle X-ray Scattering (WAXS)

The samples for WAXS analysis were dry powders obtained by drying the sample solution in a vacuum oven at 40 °C for 24 h.

#### 2.2.6. Differential Scanning Calorimetry (DSC)

The samples for DSC experiments were dry powders obtained by drying the solution of platelets in a vacuum oven at 40 °C for 24 h.

## 3. Results

The assembly procedure was simply that the P-E-P and α-CD solid (EO/α-CD = 1:1, molar ratio) were directly heated in the solvent at 80 °C for 1 h and then cooled down naturally to room temperature (r.t., 21 °C) within 3 h. Solvent mixtures from three solvents were used in this study, H_2_O, toluene, and 2-propanol (2-PrOH), since they were the good, marginal, and poor solvents for α-CD, respectively. Depending on the compositions of the solvent mixture, nanoplatelets with different shapes could be fabricated. Typically, when a mixture of H_2_O/2-PrOH (1:1, *v*/*v*) was used, exclusively regular hexagonal platelets were produced, as observed under the transmission electron microscope (TEM) and scanning electron microscope (SEM) (Figure 1e,h). The composition of the solvent mixture is critical for the final morphology. When excessive H_2_O was used, geometrically ambiguous platelets with nonuniform size were obtained. Meanwhile, when 2-PrOH was in excess, α-CD tended to form irregular aggregates (Figure S1). Additionally, our control experiments showed that both α-CD and P-E-P were crucial for the formation of regular hexagonal platelets (Figures S1 and S2). While P-E-P was completely soluble in both H_2_O and 2-PrOH, pure α-CD could not form any well-defined aggregates in the solution since it was not dispersible in the solvent mixture.

The assembly process was a typical self-nucleation and growth process. At elevated temperatures, both α-CD and P-E-P were completely dissolved in the binary solvent mixture. During the cooling process, P-E-P threaded into α-CD to form ICs, which subsequently crystallized to grow in size. Specifically, small-sized spiral hexagonal platelets were observed at 60 °C. Afterward, well-defined hexagonal platelets appeared when the solution was cooled down to 40 °C, which would continue to grow in size when the solution was further cooled down to r.t. (Figure S3).

Therefore, it could be concluded that these platelets were fabricated firstly via the formation of ICs by threading the α-CD onto the PEO block in the middle of the P-E-P triblock copolymer. The ICs subsequently tended to aggregate and crystallize. Meanwhile, the terminal PPO block would stretch into the solution from both the top and bottom sides of the crystals, limiting their growth to only two dimensions, and also stabilize the crystalline platelets, as depicted in the top row in Figure 1a.

The same procedures were adopted to prepare square platelets, except the solvent mixture was changed to H_2_O/toluene (1:1, *v*/*v*) (Figure 1c,f,i). It was noteworthy that since water and toluene are immiscible, a high stirring rate of 800 r/min was used to mix the two solvents as finely as possible during the heating process. Although they still phase-separated into two layers when the stirring was stopped, the square platelets of identical morphology and dimension were found in both aqueous and toluene layers (Figure S4). Despite the separated solvent layers, the assembly morphology was still quite sensitive to the solvent composition. When toluene content was lower (H_2_O:toluene ≥ 1:0.6), illy-defined square platelets were formed from ICs. While an excessive amount of toluene was added, the crystals appeared to be larger and rectangular (Figure S5). Interestingly, when solely α-CD was dispersed in the mixture, irregular aggregates were obtained when the toluene content was lower (H_2_O:toluene ≥ 1:0.3). With the increase in toluene content, the aggregates became more and more regular toward rectangular crystals, instead of squares (Figure S6).

Subsequently, we wondered what would happen in the tertiary solvent mixture. By keeping the H_2_O and 2-PrOH volumes equal, and adding a small portion of toluene, surprisingly, it was found that the hexagonal platelets lost their hexagonal features, and the vertices became round to obtain exclusively circular platelets at a volume ratio of 1:1:0.3 (H_2_O/isopropanol/toluene, *v*/*v*/*v*) (Figure 1d,g,j). Similarly, these circular platelets were also very sensitive to solvent compositions; when the ratio of H_2_O to toluene was fixed, only slight variation in the 2-PrOH content would lead to irregular shapes (Figure S7).

**Figure 2:**
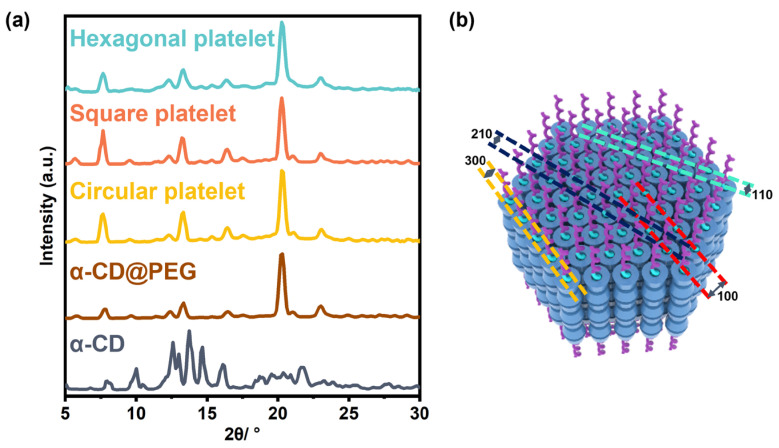
(**a**) WAXS profiles of the crystals of α-CD, α-CD@PEG, and the three inclusion complexes in the three different solvent mixtures. (**b**) The corresponding view down the *c* axis and along the ab plane of the unit cell.

To provide a mechanistic understanding of this unusual assembly process, wide-angle X-ray scattering (WAXS) experiments were conducted to characterize the crystalline structures of these nanoplatelets. Unexpectedly, despite their distinct geometrical shapes, all these platelets shared almost the same crystalline lattice packings. As shown in Figure 2a, these scattering patterns were almost identical to that from the IC of α-CD and PEG (α-CD@PEG) but were different from that of pure α-CD. The intensive peak observed at 20.0° (210) in the WAXS pattern was the characteristic peak of α-CD-based IC crystals adopting a channel-type structure [33]. Furthermore, the significant peaks at about 7.6° (100), 12.8° (110), 20.0° (210), and 22.6° (300) originated from the hexagonal packing of the α-CD-based IC crystals (Figure 2b) [34]. It can be seen from Table S1 that the lattice parameters *a* and *b* of the square platelets are slightly larger than those of the hexagonal and circular platelets by about 0.03 Å, which was probably caused by a small number of water molecules left between the outer edges of α-CD, as α-CD was known to form hydrates with water molecules due to hydrogen bonding [35]. For hexagonal platelets, H_2_O and 2-PrOH are mutually soluble as single-phase solvents, which means the hydrogen bonding between 2-PrOH and H_2_O would compete against the formation of α-CD hydrates. Moreover, as 2-PrOH is a poor solvent for α-CD, the residual water in the interspace between α-CD molecules was reduced to a certain extent. However, during the assembly process of square platelets, H_2_O and toluene separated into two layers; the easier incorporation of water molecules in the gaps between α-CD molecules resulted in a slight increase in the lattice constant of square platelets on the *ab* plane. These results suggested that the solvent composition, though vital for determining assembly morphology, would not significantly affect the crystalline molecular packing. Despite their smooth and flat surface from SEM images, screw-growth layer structures could occasionally be seen from the side view of some tilted platelets (Figure S8), suggesting the screw-dislocation growth mode of these platelets [32]. These results clearly demonstrated that the formation of these platelets should be mainly driven by the crystallization of the ICs, a process similar to the so-called “crystallization-driven self-assembly” [13,14,15,16].

**Figure 3:**
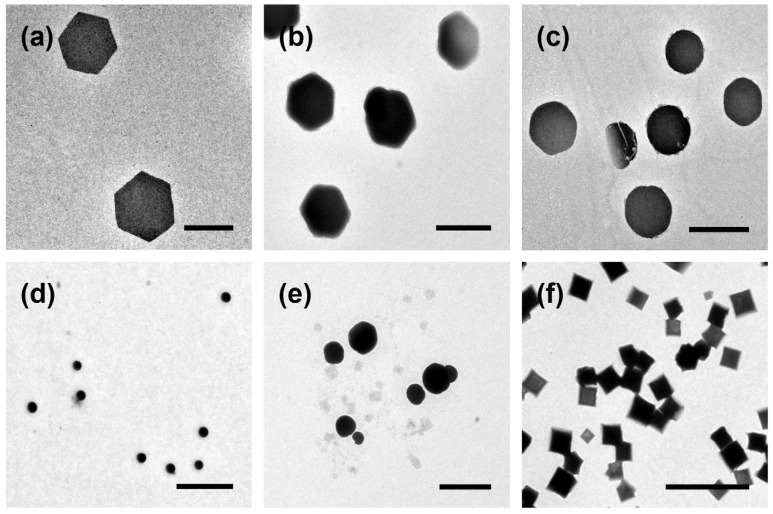
Variations of the shapes by tuning the solvent compositions. TEM images of the structures formed in (**a**) H_2_O:2-PrOH:toluene = 1:1:0; (**b**) H_2_O:2-PrOH:toluene = 1:1:0.1; (**c**) H_2_O:2-PrOH:toluene = 1:1:0.2; (**d**) H_2_O:2-PrOH:toluene = 1:1:0.3; (**e**) H_2_O:2-PrOH:toluene = 1:0.2:0.8; (**f**) H_2_O:2-PrOH:toluene = 1:0:1. Scale bars are 2 μm.

Consequently, a plausible reason for the formation of different shapes was that these solvent molecules could strongly influence the growth rates of crystals in different directions [36], thus leading to the formation of platelets with different shapes [37]. In order to further prove the influence of solvent composition on the shape of these platelets, the solvent composition was systematically adjusted. By switching the solvent composition from H_2_O:2-PrOH:toluene = 1:1:0 to 1:1:0.1 and 1:1:0.2 followed by the same heating–cooling assembly procedure, interestingly, the hexagonal platelets gradually lost their geometrical features and transformed towards circular shapes (Figure 3a–c). Moreover, when the toluene content increased and the 2-PrOH content decreased to reach a volume ratio of H_2_O:2-PrOH:toluene = 1:0.2:0.8, the circular platelets gradually recovered their geometrical features but formed square platelets instead (Figure 3d–f).

It can be seen from their TEM images that the platelets of each shape are highly uniform. The polydispersity of the areas (PDI_area_) of these platelets was defined as the ratio of weight average area over the number average area to evaluate their uniformity. The PDI values of these platelets, shown in Figure 1, were determined to be 1.01, suggesting a remarkable uniformity in size. In addition, the dynamic light scattering (DLS) experiments of the three geometrical nanoplatelets illustrated the colloidal stability of these nanoplatelets in solution, and further proved the size uniformity of the IC nanoplatelets (Figure S9). Subsequently, an interesting question is raised as to whether we can control their sizes.

**Figure 4:**
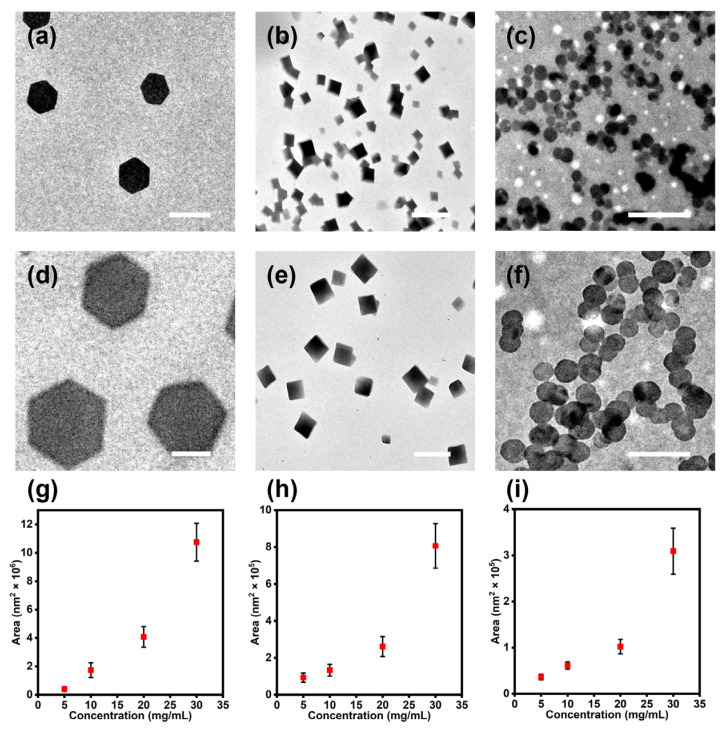
TEM images of the (**a**,**d**) hexagonal platelets, (**b**,**e**) square platelets, and (**c**,**f**) circular platelets with an α-CD concentration (EO/α-CD = 1:1, molar ratio) of (**a**–**c**) 5 mg/mL and (**d**–**f**) 20 mg/mL. (**g**–**i**) Variation of nanoplatelet sizes versus the α-CD concentrations of (**g**) hexagonal platelets, (**h**) square platelets, and (**i**) circular platelets. Scale bars are 1 μm.

Surprisingly, we found a counterintuitive concentration dependence for their platelet sizes. According to the classical crystallization theories, lower supersaturation concentration prefers continuous growth instead of self-nucleation, and usually results in larger crystals [38]. However, in the current study, we found that higher concentrations led to larger platelets for all three cases. All three types of platelets were assembled at α-CD concentrations of 5, 10, 20, and 30 mg/mL (EO/α-CD = 1:1, molar ratio). The obtained geometrical platelets were all well-defined (Figure 4a–f and Figures S10–S12) and quite uniform (PDI_area_ ≤ 1.04, Tables S2–S4). The averaged area values of these platelets were plotted versus the α-CD concentration in Figure 4g–i, suggesting super-linear relationships between the area and concentration. This unusual result could possibly be attributed to the slowly decreasing solubility of ICs during the cooling process. Therefore, despite the high overall concentration, a low supersaturation was retained during the cooling process, leading to the continuous growth of crystal sizes and eventually large platelets. This claim was supported by our observation that when the solution was quenched from 80 °C to r.t., the high supersaturation led to the random self-nucleation and poor crystalline packings of the ICs, yielding illy-defined platelets (Figure S13).

Another thing worth noting was that the hexagonal platelets were significantly larger than the other two. With a fixed α-CD concentration of 20 mg/mL (EO/α-CD = 1:1, molar ratio), the average area of these assembled hexagonal platelets could reach 4.08 ± 0.71×10^6^ nm^2^, which was significantly larger than that of square platelets (2.61 ± 0.53×10^5^ nm^2^), and which was also larger than that of circular platelets (1.02 ± 0.15×10^5^ nm^2^). This could be due to the different solubility of the ICs in the three solvents. As mentioned above, H_2_O, toluene, and 2-PrOH were the good, marginal, and poor solvents for α-CD, respectively. Therefore, when the 2-PrOH content was high (H_2_O/2-PrOH = 1:1 for hexagonal platelets), the ICs were not as perfectly dissolved, and the concentration of dispersed ICs was low. When the poor solvent 2-PrOH was replaced by the marginal solvent toluene, the overall solubility of ICs was improved, and more ICs could be dissolved in the solution individually, resulting in smaller sizes for the square platelets. Moreover, when the binary mixture was changed to the tertiary mixture (H_2_O/2-PrOH/toluene = 1:1:0.3), the solubility of the ICs was further increased, leading to even smaller sizes for the circular platelets.

**Figure 5:**
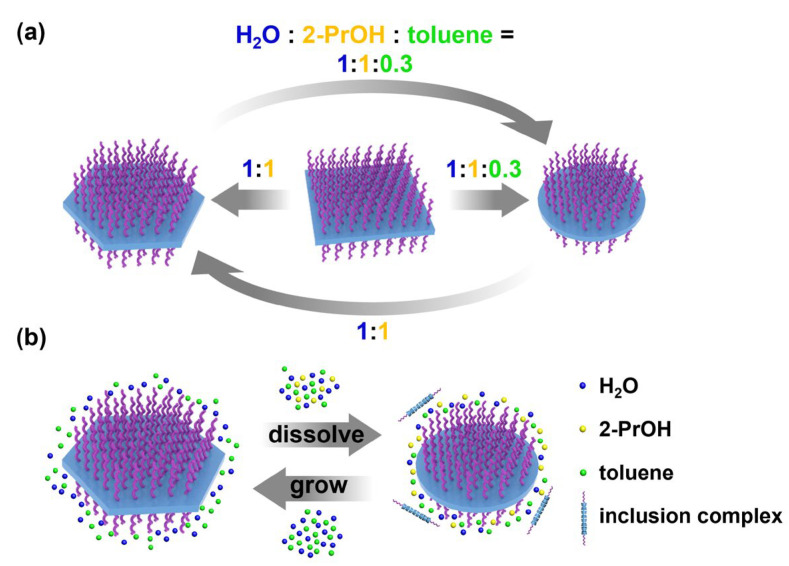
Schematic illustration of (**a**) the interconversion between different platelets and (**b**) molecular-level mechanistic illustration of the interconversion.

Based on the solvent effects on the shapes and sizes of these platelets mentioned above, we found that by simply switching the solvent composition at r.t., these platelets could be interconverted into each other without heating–cooling procedures (Figure 5a). By adding the desired amount of toluene into the binary mixture solution of H_2_O and 2-PrOH, the initial hexagonal platelets were transformed into circular platelets exclusively, as shown in Figure S13a,b. All the interconversion routes were explored (Figure 5a), and it was found that the square platelets could be converted into both hexagonal and circular platelets, which could also be interconverted into each other (Figures S14 and S15). However, neither the hexagonal nor circular platelets could be converted back to square platelets.

A cartoon depiction of the interconversion between hexagonal and circular platelets was included in Figure 5b. When the solvent composition is changed, due to the increased solubility of the ICs, they partially dissolve out from the crystalline platelets into the solution, yielding circular platelets. This solvent composition-induced transformation does not involve the complete dissolution of the IC crystals or the nucleation-growth process. Instead, it occurs through the partial dissolution of the ICs (from hexagonal or square platelets to circular ones), or the growth of the dissolved ICs onto the original platelets (from square or circular platelets to hexagonal ones). Therefore, the total number of platelets remains the same, but their sizes will change during the solvent composition variation. This speculation was confirmed, as shown in Figure S13, by the fact that when square platelets were converted into hexagonal or circular platelets, their sizes would increase or decrease, respectively. Regarding the failures in converting hexagonal or circular platelets into square platelets, it might be attributed to the lower crystalline orderliness and stability of these square platelets, which could be seen from their significantly lower melting enthalpy values obtained via differential scanning calorimetry (DSC) characterizations (Figure S16).

## 4. Conclusions

Herein, we presented the controllable assembly of different geometrical nanoplatelets from the same supramolecular subunits by simply controlling the compositions of the solvent mixture. The ICs from α-CD and PPO-*b*-PEO-*b*-PPO were used as the building block to form three different geometrical platelets, hexagonal, square, and circular, driven by the crystallization of the Ics. Their geometrical shapes were highly sensitive to solvent compositions. Interestingly, the sizes of these platelets, regardless of their shapes, were quite uniform and would increase accordingly with the overall solution concentration. Lastly, we showed that these nanoplatelets could be interconverted into each other by simply adjusting the solvent composition at room temperature via the partial dissolution of the ICs. We believe the current study has demonstrated an easy yet highly efficient method for fabricating two-dimensional nano-objects with controllable geometrical shapes and sizes, opening a novel avenue for the design and construction of nanostructures with broad accessibility and utility.

Extending this method to other macrocyclic molecular systems, such as cucurbituril and pillararene, led to more shapes of nanoplatelets are expected. Furthermore, the different geometrical nanoplatelets can hopefully be applied in the fields of aggregation-induced luminescence (AIE), catalysis, and biological imaging. The variation in aggregate morphology will greatly affect its AIE characteristics; therefore, two-dimensional materials with different fluorescence intensity can be constructed through the inclusion between cyclodextrin and block copolymers with AIE characteristics. Alternatively, two-dimensional nanoplatelets prepared in this article have a large specific surface area, which is expected to be applied in the catalytic field by labeling the PPO block with catalysts. Lastly, the IC nanoplatelets constructed in this paper prepared with α-CD and P-E-P are all biocompatible, which are expected to be applied in the field of biological imaging via modifying the PPO blocks with fluorescent molecules.

## Data Availability

Original data are available when requested.

## Supplementary Materials

The following supporting information can be downloaded at: https://www.mdpi.com/article/10.3390/polym15112547/s1, Figure S1: TEM images of the assembled products from 𝛼-CD and P-E-P in a mixture of H_2_O and 2-PrOH with different ratio. The ratio of H_2_O and 2-PrOH are (a) 1:0; (b) 1:0.6; (c) 1:0.8; (d) 1:1; © 1:2; and (f) 0:1. Scale bars are 1 μm; Figure S2: TEM images of the products generated by 𝛼-CD in different solutions at 80 °C for 1 h, cooling down naturally. (a) H_2_O; (b) 2-PrOH; (c) 2-PrOH:H_2_O = 1:1. Scale bars are 1 μm; Figure S3: TEM images of the natural cooling process of hexagonal platelets from 80 °C to (a) 60 °C; (b) 40 °C; and (c) r.t. Scale bars are 1 μm; Figure S4: TEM images of the square platelets from (a) aqueous layer; and (b) toluene layer. Scale bars are 1 μm; Figure S5: TEM images of the assembled platelets from 𝛼-CD and P-E-P in a mixture of H_2_O and toluene with different ratio. The ratio of H_2_O and toluene are (a) 1:0.2; (b) 1:0.6; (c) 1:1; and (d) 1:3. Scale bars are 1 μm; Figure S6: TEM images of the assembled products from 𝛼-CD in a mixture of H_2_O and toluene with different ratio. The ratio of H_2_O and toluene are (a) 1:0; (b) 1:0.2; (c) 1:0.3; (d) 1:0.6;(e) 1:0.8; and (f) 1:1. Scale bars are 1 μm; Figure S7: Variations of the shapes by tuning the solvent compositions slightly. TEM images of the structures formed in (a) H_2_O:2-PrOH:toluene = 1:1:0.4; (b) H_2_O:2-PrOH:toluene = 1:1:0.5; (c) H_2_O:2-PrOH:toluene = 1:0.9:0.3; (d) H_2_O:2-PrOH:toluene = 1:1.1:0.3. Scale bars are 1 μm; Figure S8: TEM images of the screw dislocation structure of (a) hexagonal platelets; (b) square platelets. Scale bars are 1 μm; Figure S9: DLS data of the hexagonal, square, and circular platelets; Figure S10: TEM images of the hexagonal platelets with the concentration of (a) 5 mg/mL; (b) 10 mg/mL; (c) 20 mg/mL; (d) 30 mg/mL. Scale bars are 1 μm; Figure S11: TEM images of the square platelets with the concentration of (a) 5 mg/mL; (b) 10 mg/mL; (c) 20 mg/mL; (d) 30 mg/mL. Scale bars are 1 μm; Figure S12: TEM images of the circular platelets with the concentration of (a) 5 mg/mL; (b) 10 mg/mL; (c) 20 mg/mL; (d) 30 mg/mL. Scale bars are 1 μm; Figure S13: TEM images of the illy-defined platelets caused by quenching from 80 °C to r.t.. Scale bars are 2 μm; Figure S14: TEM images of (a) square platelets; (b) the hexagonal platelets transformed from (a); and (c) the circular platelets transformed from (a). Scale bars are 1 μm; Figure S15: TEM images of (a) hexagonal platelets; (b) the circular platelets transformed from (a); (c) circular platelets; and (d) the hexagonal platelets transformed from (c). Scale bars are 1 μm; Figure S16: DSC curve of (a) hexagonal platelets; (b) square platelets; (c) circular platelets; Table S1: Lattice parameters and cell volume of hexagonal, square and circular platelets; Table S2: Contour side length distributions and area distributions of P-E-P/𝛼-CD hexagonal platelets; Table S3. Contour side length distributions and area distributions of P-E-P/𝛼-CD square platelets; Table S4. Area distributions of P-E-P/𝛼-CD circular platelets.
