# Supplementary material for: Assembly of Supramolecular Nanoplatelets with Tailorable Geometrical Shapes and Dimensions
Source: Polymers (Basel). 2023 May 31;15(11):2547. doi: 10.3390/polym15112547 (PMC10255392; doi:10.3390/polym15112547)
Supplement: Supplementary file 1 [file polymers-15-02547-s001.zip › polymers-2425522-supplementary.pdf]

Supplementary Materials for

Assembly of Supramolecular Nanoplatelets with Tailorable Geometrical Shapes and Dimensions

Differential scanning calorimetry (DSC)

Differential scanning calorimetry (DSC) were performed on a TA Instruments Q100 calorimeter at a scan rate of 10 °C/min under nitrogen (50 mL/min).

Wide-angle X-ray scattering (WAXS)

Wide-angle X-ray scattering (WAXS) analysis was implemented by employing Ganesha system (SAXSLAB, U.S) equipped with a multilayer focused Cu Kα radiation as the X-ray source (Genix3D Cu ULD) and a semiconductor detector (Pilatus 300 K, DECTRIS, Swiss). The system was operated at 50 kV and 0.6 mA.

Transmission Electron Microscopy (TEM)

The samples for electron microscopy were prepared by drop-casting one drop (~ 7 μL) of the solution onto a carbon coated copper grid (Beijing Zhongjingkeyi Technology Co., Ltd, mesh 230). Grids were placed on a piece of filter paper in advance to quickly remove excess solvent in 1 s to prevent further morphological change. Bright-field transmission electron microscopy (TEM) micrographs were obtained on a JEM1200EX microscope operating at 100 kV. No staining was applied for TEM samples. Images were analyzed using the Image-Pro Plus 6.0 software, which is free and available online. As the platelets reported here are trapped kinetically because of the crystallization of α-cyclodextrin, the morphologies observed from dried samples by TEM are anticipated to match closely those observed in solution. For the statistical length analyses, more than 200 platelets in several images were analyzed with the software in order to obtain the area information. The number average geometrical platelet (hexagonal platelet, square platelet and circular platelet) side length (L_n) and weight average geometrical platelet side length (L_w) were calculated using Eqs. 1 and 2 (L_i, the contour lengths; N_i, the number of length L_i; n, the number of

$$(1) L_n = \frac{\sum_{i=1}^{n} N_i L_i}{\sum_{i=1}^{n} N_i} \quad (2) L_w = \frac{\sum_{i=1}^{n} N_i {L_i}^2}{\sum_{i=1}^{n} N_i L_i} \quad (3) A_n = \frac{\sum_{i=1}^{n} N_i A_i}{\sum_{i=1}^{n} N_i} \quad (4) A_w = \frac{\sum_{i=1}^{n} N_i {A_i}^2}{\sum_{i=1}^{n} N_i A_i}$$

lengths examined in each sample). The standard deviations (σ) of the measured side lengths were calculated directly from the data set.

Scanning Electron Microscopy (SEM)

SEM experiments were conducted directly on the carbon-coated copper grid used for TEM analysis. SEM images were recorded by using a Hitachi SU8020 microscope operating at 30kV. An ultrathin coating of Au (~ 5 nm) was deposited via high vacuum evaporation.

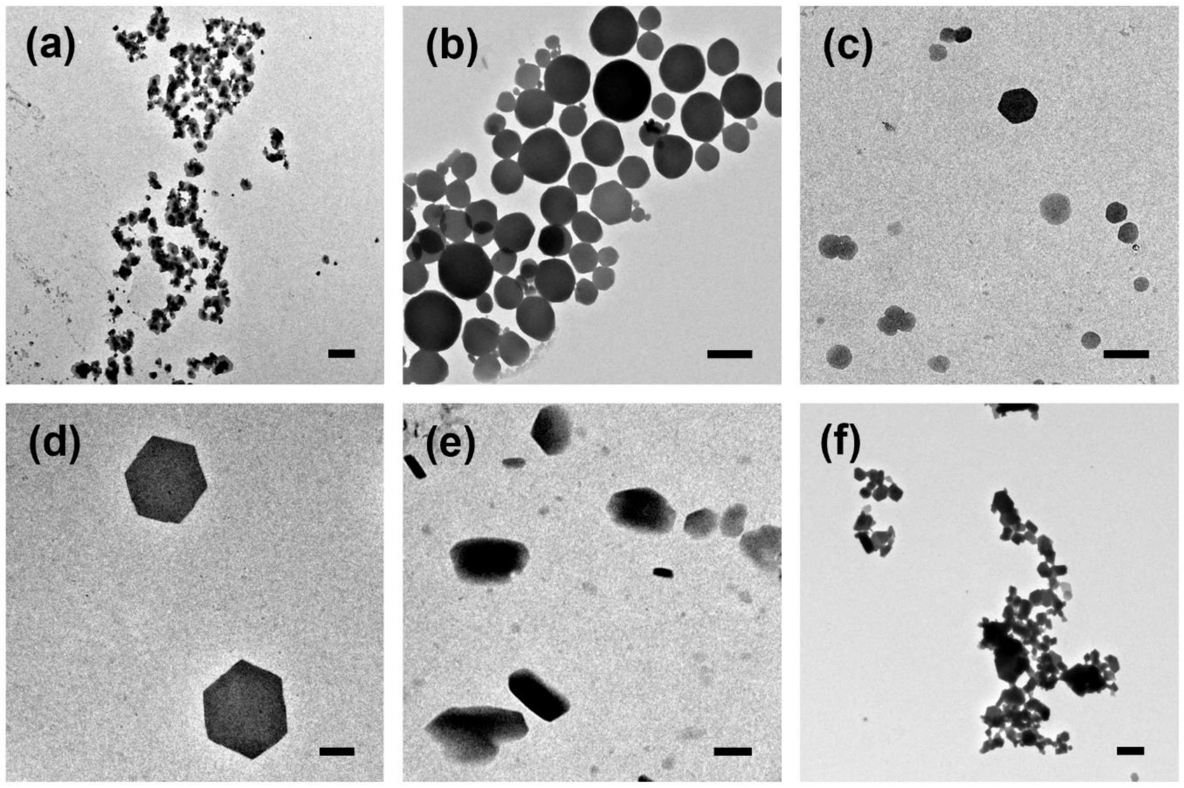

Figure S1. TEM images of the assembled products from α-CD and P-E-P in a mixture of H_2O and 2-PrOH with different ratio. The ratio of H_2O and 2-PrOH are (a) 1 : 0 ; (b) 1 : 0.6 ; (c) 1 : 0.8; (d) 1 : 1; (e) 1 : 2; and (f) 0 : 1. Scale bars are 1 μm.

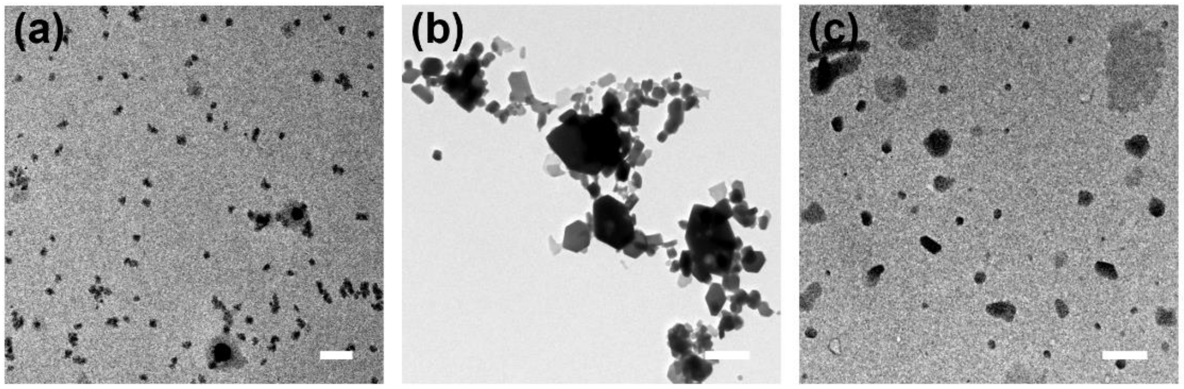

Figure S2. TEM images of the products generated by α-CD in different solutions at 80 ^{o}C for 1 h, cooling down naturally. (a) H_2O; (b) 2-PrOH; (c) 2-PrOH : H_2O = 1 : 1. Scale bars are 1 μm.

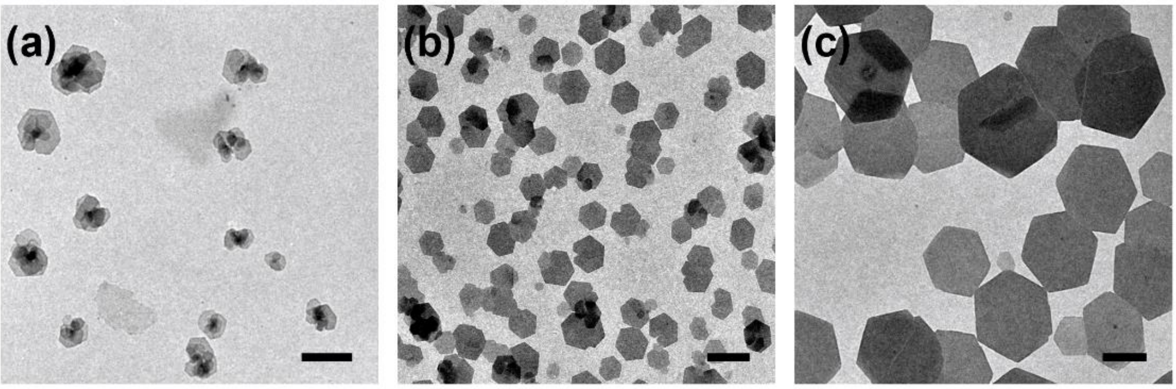

Figure S3. TEM images of the natural cooling process of hexagonal platelets from 80 ^{o}C to (a) 60 ^{o}C; (b) 40 ^{o}C; and (c) r.t.. Scale bars are 1 μm.

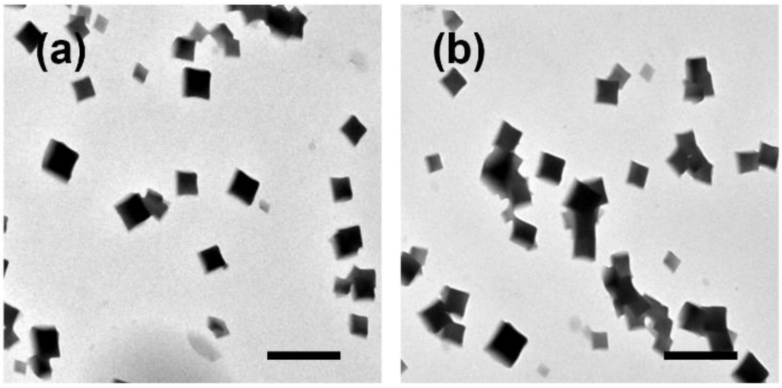

Figure S4. TEM images of the square platelets from (a) aqueous layer; and (b) toluene layer. Scale bars are 1 μm.

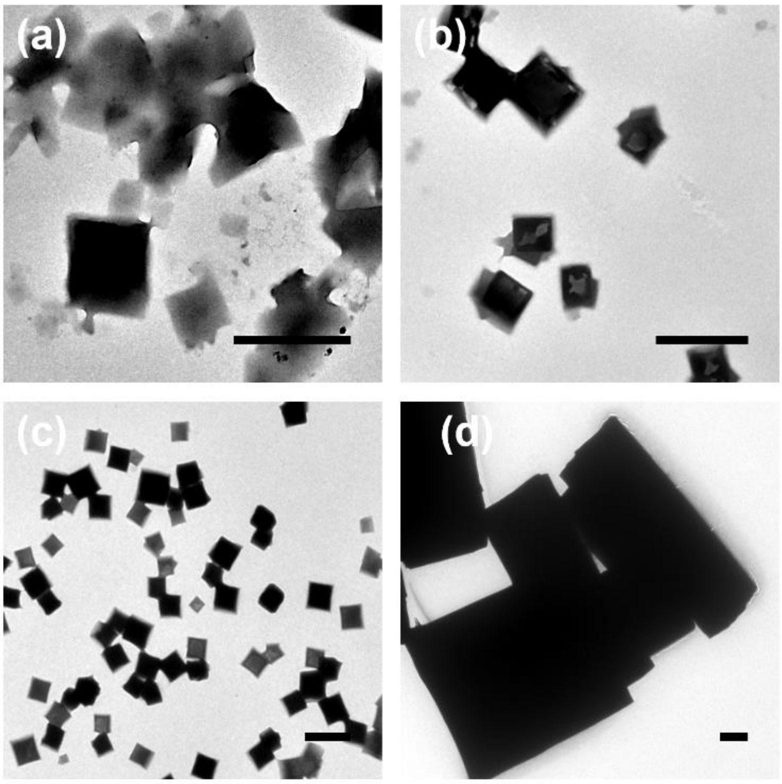

Figure S5. TEM images of the assembled platelets from α-CD and P-E-P in a mixture of H_2O and toluene with different ratio. The ratio of H_2O and toluene are (a) 1 : 0.2 ; (b) 1 : 0.6 ; (c) 1 : 1; and (d) 1 : 3. Scale bars are 1 μm.

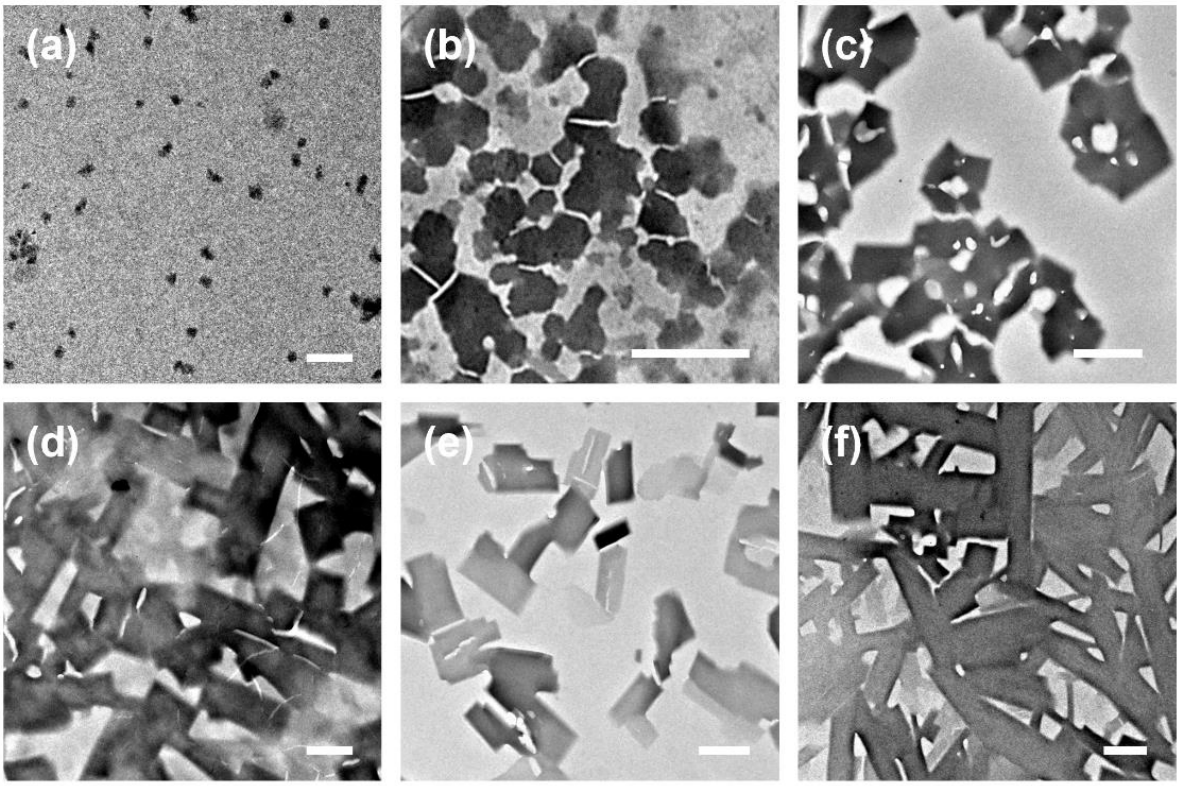

Figure S6. TEM images of the assembled products from α-CD in a mixture of H_2O and toluene with different ratio. The ratio of H_2O and toluene are (a) 1 : 0 ; (b) 1 : 0.2 ; (c) 1 : 0.3 ; (d) 1 : 0.6 ;(e) 1 : 0.8 ; and (f) 1 : 1. Scale bars are 1 μm.

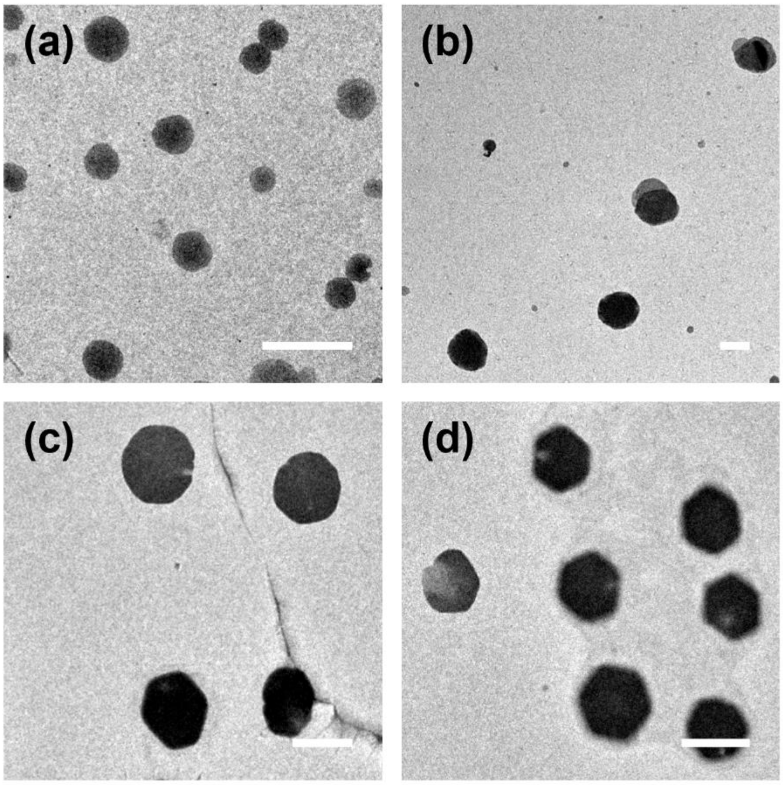

Figure S7. Variations of the shapes by tuning the solvent compositions slightly. TEM images of the structures formed in (a) H_2O : 2-PrOH : toluene = 1 : 1 : 0.4; (b) H_2O : 2-PrOH : toluene = 1 : 1 : 0.5; (c) H_2O : 2-PrOH : toluene = 1 : 0.9 : 0.3; (d) H_2O : 2-PrOH : toluene = 1 : 1.1 : 0.3. Scale bars are 1 μm.

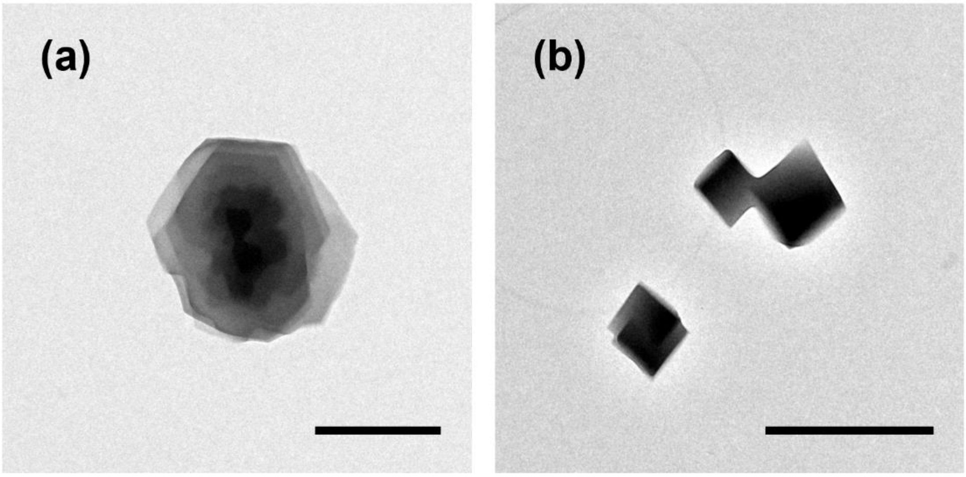

Figure S8. TEM images of the screw dislocation structure of (a) hexagonal platelets; (b) square platelets. Scale bars are 1 μm.

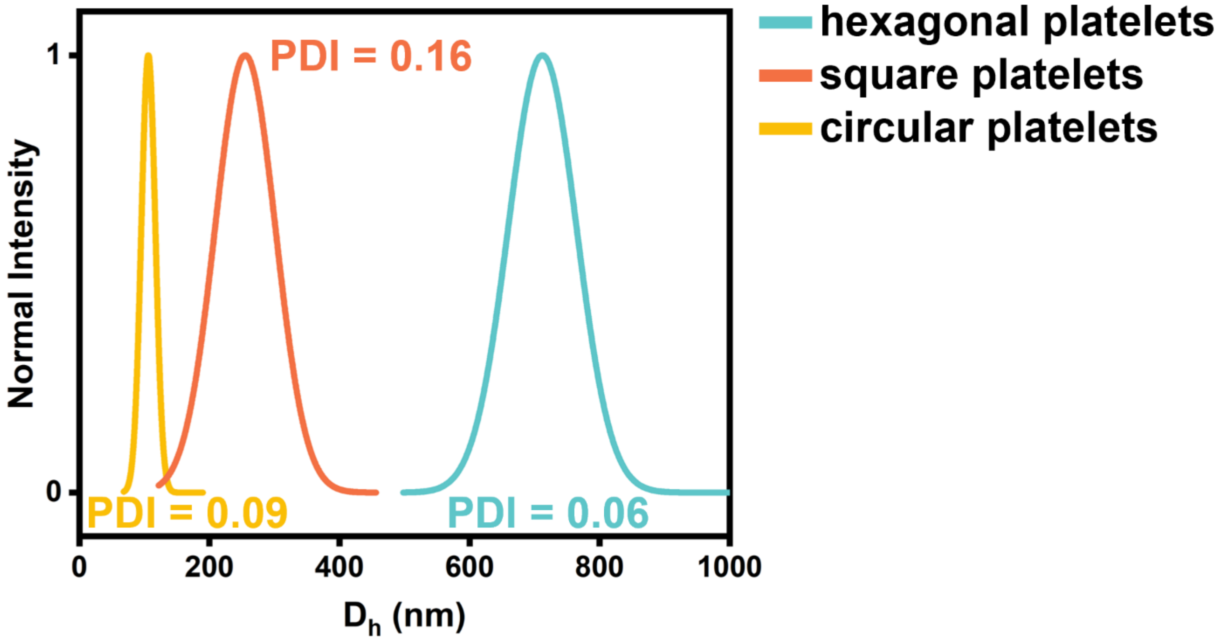

Figure S9. DLS data of the hexagonal, square and circular platelets.

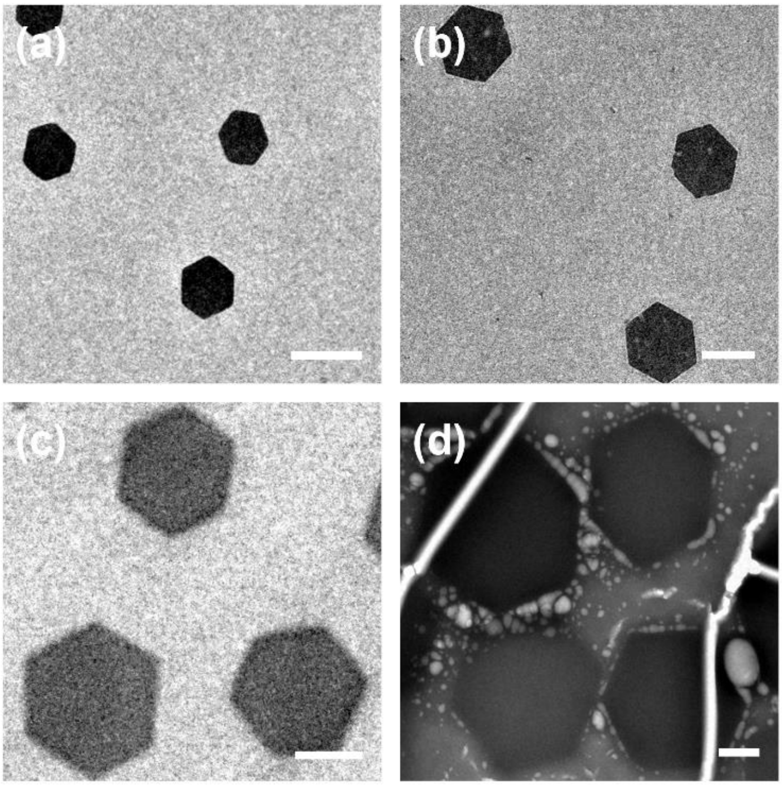

Figure S10. TEM images of the hexagonal platelets with the concentration of (a) 5 mg/mL; (b) 10 mg/mL; (c) 20 mg/mL; (d) 30 mg/mL. Scale bars are 1 μm.

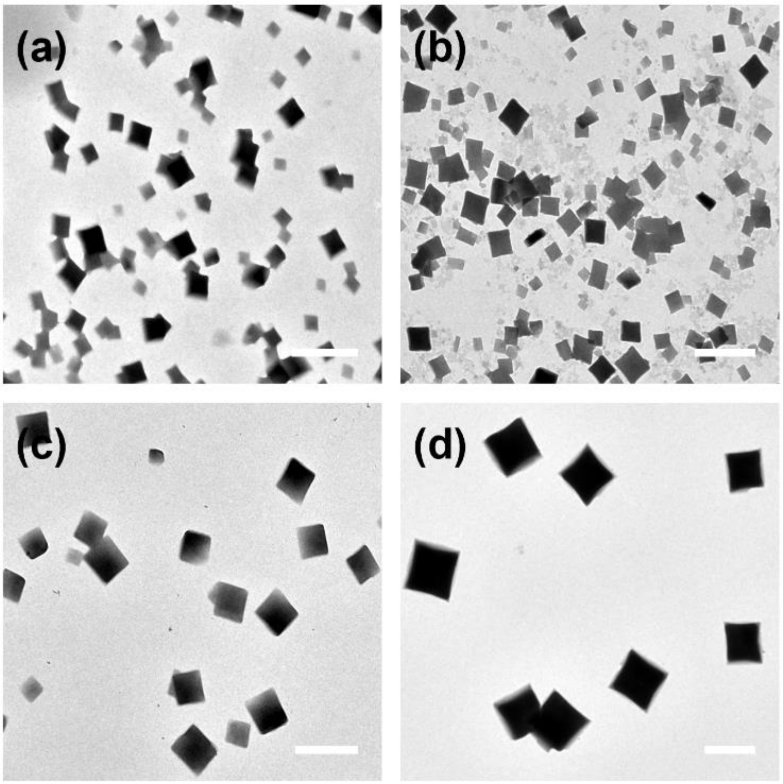

Figure S11. TEM images of the square platelets with the concentration of (a) 5 mg/mL; (b) 10 mg/mL; (c) 20 mg/mL; (d) 30 mg/mL. Scale bars are 1 μm.

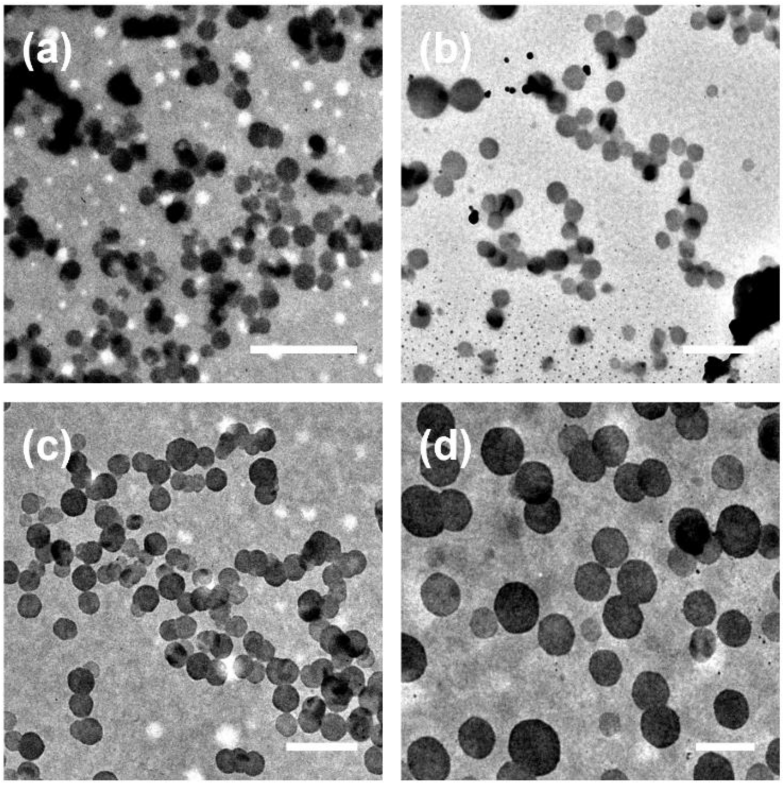

Figure S12. TEM images of the circular platelets with the concentration of (a) 5 mg/mL; (b) 10 mg/mL; (c) 20 mg/mL; (d) 30 mg/mL. Scale bars are 1 μm.

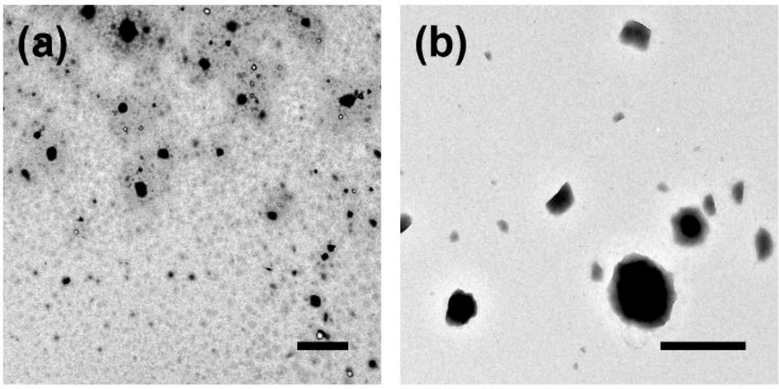

Figure S13. TEM images of the illy-defined platelets caused by quenching from 80 ^{o}C to r.t.. Scale bars are 2 μm.

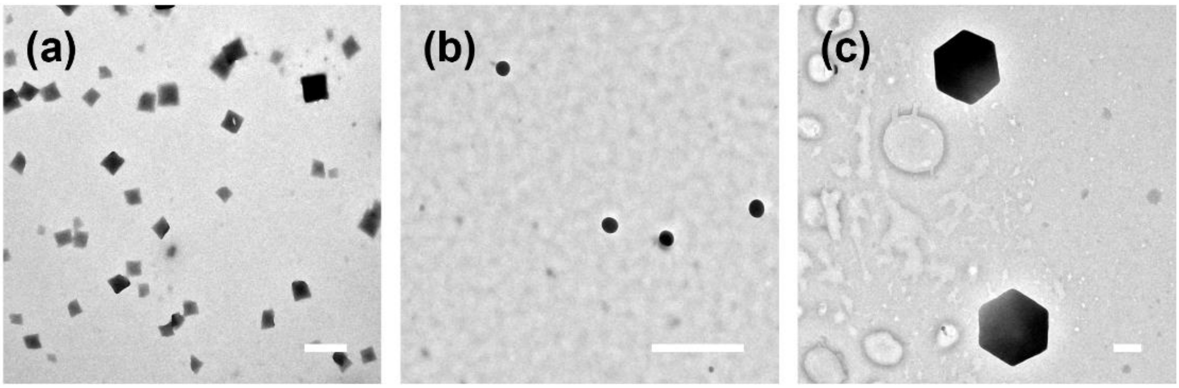

Figure S14. TEM images of (a) square platelets; (b) the hexagonal platelets transformed from (a); and (c) the circular platelets transformed from (a). Scale bars are 1 μm.

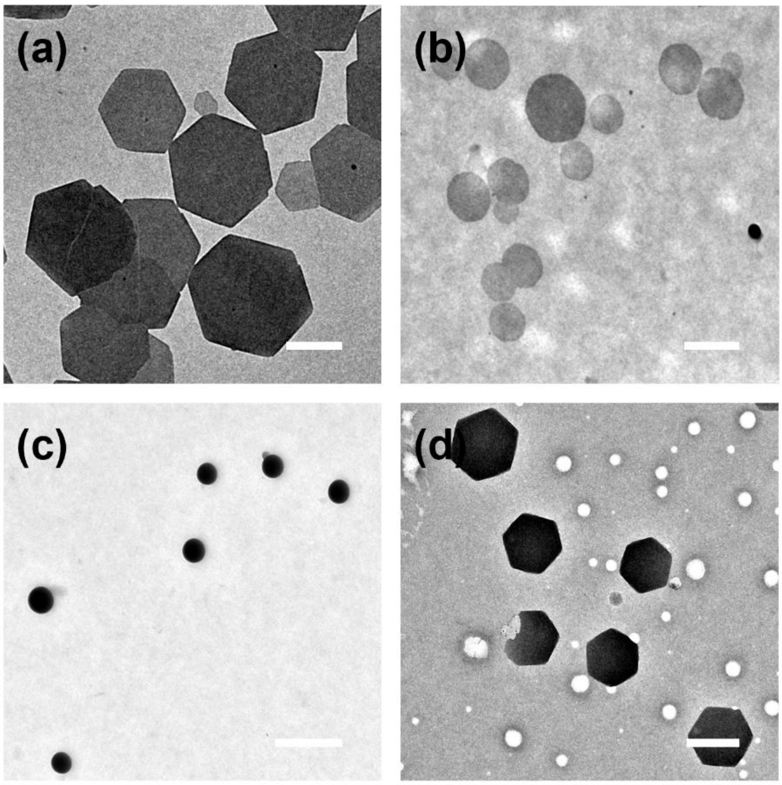

Figure S15. TEM images of (a) hexagonal platelets; (b) the circular platelets transformed from (a); (c) circular platelets; and (d) the hexagonal platelets transformed from (c). Scale bars are 1 μm.

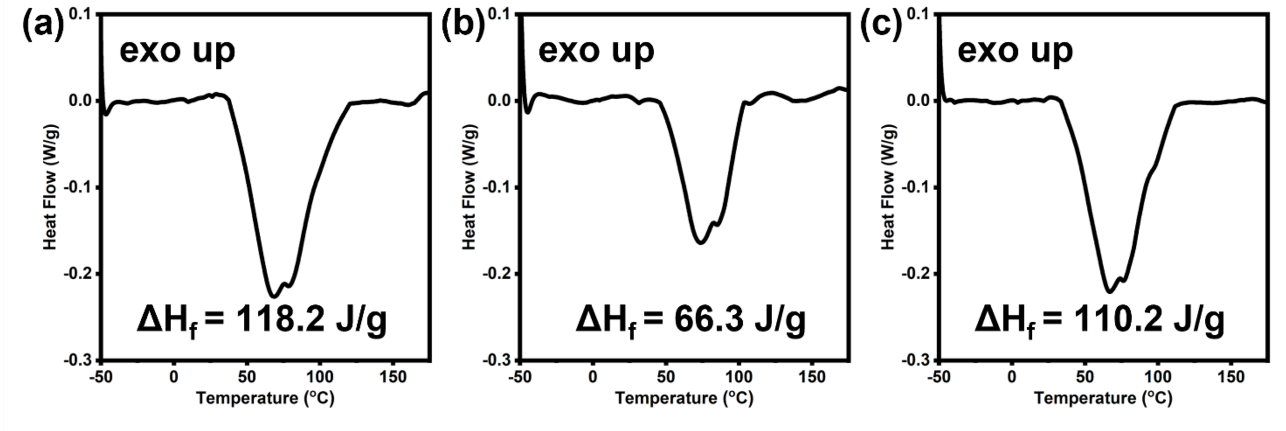

Figure S16. DSC curve of (a) hexagonal platelets; (b) square platelets; (c) circular platelets.

Table S1. Lattice parameters and cell volume of hexagonal, square and circular platelets.

	crystal system	a,b (Å)	c (Å)	V (Å^3)
hexagonal platelets	hexagonal	13.3348	7.1959	1109.8
square platelets	hexagonal	13.3698	7.1959	1113.9
circular platelets	hexagonal	13.3305	7.1959	1107.4

Table S2. Contour side length distributions and area distributions of P-E-P/α-CD hexagonal platelets.

$c_{\alpha\text{-}CD}$ (mg/mL)	L_n (nm)	L_w (nm)	L_w/L_n	σ (nm)	σ/L_n	A_n (×10^6 nm^2)	A_w (×10^6 nm^2)	A_w/A_n	σ (×10^6 nm^2)	σ/A_n
5	393	397	1.01	73	0.19	0.41	0.42	1.02	0.07	0.03
10	808	825	1.02	118	0.15	1.73	1.78	1.03	0.29	0.17
20	1248	1257	1.01	218	0.17	4.08	4.19	1.02	0.69	0.17
30	2031	2037	1.00	242	0.12	10.75	10.90	1.01	1.28	0.12

Table S3. Contour side length distributions and area distributions of P-E-P/α-CD square platelets.

$c_{\alpha\text{-}CD}$ (mg/mL)	L_n (nm)	L_w (nm)	L_w/L_n	σ (nm)	σ/L_n	A_n (×10^5 nm^2)	A_w (×10^5 nm^2)	A_w/A_n	σ (×10^5 nm^2)	σ/A_n
5	303	308	1.02	40	0.13	0.93	0.97	1.04	0.22	0.23
10	362	367	1.01	43	0.12	1.33	1.37	1.03	0.31	0.23
20	508	514	1.01	52	0.10	2.61	2.72	1.04	0.53	0.20
30	896	900	1.01	66	0.07	8.06	8.23	1.02	1.17	0.15

Table S4. Area distributions of P-E-P/α-CD circular platelets.

$c_{\alpha\text{-}CD}$ (mg/mL)	A_n ($\times 10^5$ nm^2)	A_w ($\times 10^5$ nm^2)	A_w/A_n	σ ($\times 10^5$nm^2)	σ/A_n
5	0.36	0.37	1.03	0.06	0.17
10	0.61	0.62	1.02	0.08	0.13
20	1.02	1.05	1.02	0.15	0.15
30	3.09	3.16	1.02	0.49	0.16
